# Simultaneous measurements of knee motion using an optical tracking system and radiostereometric analysis (RSA)

**DOI:** 10.3109/17453674.2011.570675

**Published:** 2011-04-05

**Authors:** Roy Tranberg, Tuuli Saari, Roland Zügner, Johan Kärrholm

**Affiliations:** ^1^Department of Orthopaedics, Sahlgrenska University Hospital, Mölndal; ^2^Lundberg Laboratory for Orthopaedic Research, Department of Orthopaedics, Institute of Clinical Sciences, Sahlgrenska Academy, University of Gothenburg, Sahlgrenska University Hospital, Göteborg, Sweden

## Abstract

**Background and purpose:**

Invasive methods are more reproducible and accurate than non-invasive ones when it comes to recording knee kinematics, but they are usually less accessible and less safe, mainly due to risk of infection. For this reason, non-invasive methods with passive markers are widely used. With these methods, varying marker sets based on a number of single markers, or sets of markers, known as clusters, are used to track body segments. We compared one invasive method—radiostereometric analysis—with a non-invasive method, an optical tracking system with 15 skin-mounted markers.

**Methods:**

9 subjects (10 knees) were investigated simultaneously with a dynamic RSA system and a motion-capture system while performing an active knee extension.

**Results:**

For flexion/extension, there was good agreement on an individual basis and at the group level. For internal/external rotation, the group mean was fairly similar, up to 25 degrees of flexion. Recordings of abductions and/or adductions revealed a systematic mean difference of 2–4 degrees during the range of flexion measured. The correlation between the 2 methods in the horizontal and frontal planes was poor.

**Interpretation:**

Our skin-marker model provided reliable data for flexion/extension. Recordings of internal/external rotation and abduction/adduction were less accurate on an individual basis than at the group level, most probably due to soft-tissue motion and the presence of small true motion in these planes.

Techniques used to record joint kinematics can either be invasive or non-invasive. Invasive methods rely on devices or markers fixed to the skeleton, which means that they become more reproducible and describe more accurately the motions that occur. Radiostereometric analysis is one such invasive method with detailed documentation (Kärrholm et al. 1997, Valstar et al. 2005, Bragdon et al. 2006). Other invasive methods use cortical bone pins (Benoit et al. 2006) or transducers activated during a surgical procedure (Beynnon and Fleming 1998), which limits their applicability to a short time period due to the risk of infection.

All methods that require penetration of the skin involve a risk of complications. For this reason, non-invasive techniques are used most frequently in clinical practice. All systems, whether invasive or non-invasive, require the determination of each body segment of interest involved in the joint motion that is being studied.

In non-invasive methods, passive markers consisting of reflective spheres are used. These markers are commonly attached to the skin with double-sided adhesive tape. To record the 3-dimensional position of markers, a set of 2 or several infra-red video cameras are used. Varying marker sets based on a number of single markers attached to the skin, or sets of markers placed on a plastic shell—known as clusters—and used to track each body segment. The question of whether marker sets based on clusters or single skin markers should be used has been discussed. Ferrari et al. (2008) compared 5 currently used marker sets for gait analysis. They concluded that high correlations could be observed between all protocols marker sets. However, tracking of one or more bone segments with this technique is associated with errors caused by soft-tissue artifacts (Karlsson and Tranberg 1999). Several methods have been suggested to map out and reduce these artifacts (Ramsey and Wretenberg 1999, Stagni et al. 2005, Schache et al. 2006).

Furthermore, Lucchetti and co-authors (1998) showed that the effects of these artifacts could be reduced by introducing a compensating algorithm into the calculations. However, irrespective of the marker model used, the extent to which these systems reproduce the actual joint angles under study remains uncertain.

To investigate this issue, we studied patients during active knee extension, which was recorded simultaneously with both an optical tracking system based on the Lundberg skin-marker model (Weidow et al. 2006) and a dynamic radiostereometric analysis system (Saari et al. 2005).

## Patients and methods

9 subjects (7 females) who had undergone total knee arthroplasty (TKA) were studied (Table). During the operation, 5–7 tantalum markers with a diameter of 0.8 mm were inserted into the tibia and femur. 7 subjects were studied after 1 year and 2 subjects after 2 years.

**Table:** Descriptive characteristics of participants

Subject	Sex	Side	Age	Height	Weight	BMI
			(years)	(m)	(kg)	(kg/m^2^)
1	F	Right	62	1.72	80	27.04
2	F	Right	65	1.66	94	34.11
3a	F	Left	59	1.78	75	23.67
3b	F	Right	59	1.78	75	23.67
4	M	Right	72	1.74	90	29.73
5	F	Left	63	1.73	105	35.08
6	F	Left	61	1.76	116	37.45
7	F	Right	63	1.62	60	22.86
8	F	Left	70	1.60	83	32.46
9	M	Left	63	1.75	75	24.49

We used dynamic RSA with 2 film exchangers placed parallel to each another. The 2 film exchangers were set to expose in an order of 4-4-3-3-2-2 exposures per second. A uniplanar calibration cage (RSA Biomedical, Umeå, Sweden) was attached in front of the film exchangers. Both X-ray tubes were placed symmetrically, with a film-focus distance of 1.5 m and at an angle of 20 degrees in relation to an axis perpendicular to the calibration cage.

An optical tracking system (OTS) consisting of 8 cameras (MCU 240, Qualisys AB, Göteborg, Sweden), was used to record skin-marker positions. Cameras were placed to surround the subject without interfering with the X-ray equipment. Dynamic calibration was then performed, resulting in a total measurable volume of 2.4 m^3^ (1.6 × 1.0 × 1.5 m). After the calibration of the OTS system was complete, a static recording of the position of the RSA calibration cage was made to obtain the systematic difference between the two coordinate systems.

Before the dynamic RSA examination, we performed a static RSA examination with the subject in supine position. In this exposure, the knee was aligned with the RSA cage coordinate system in a standardized way (Saari et al. 2003). The position of the knee in this examination constituted the “starting” or calibration position for the subsequent dynamic RSA measurements. In order to record the knee kinematics, 15 spherical reflective markers with a diameter of 19 mm were attached to the skin with double-sided adhesive tape. Markers were attached to the skin on the sacrum, anterior superior iliac spine, lateral knee-joint line, proximal to the superior border of the patella, tibial tubercle, heel, lateral malleolus, and between the second and third metatarsals (Weidow et al. 2006). A physiotherapist with more than 10 years of experience performed all marker attachments on all subjects. The subjects were then asked to enter the measurement volume and a static reference recording was obtained with the OTS while the subjects were standing upright, aligned with the x-axis of the global co-ordinate system. Prior to the simultaneously recorded measurement, all subjects were instructed to stand with their knee in what was for them a maximum flexed position and, at a given signal, to slowly extend their knee as much as possible.To ensure that both systems were recording at the same time, opto-sound synchronization was used. This made it possible to synchronize each measurement within 0.04 s.

The stereo radiographs were scanned (Scan Maker 9800XL; Microtek International, Taiwan) and each of the cage and patient markers was measured using digitized images. We evaluated knee motions using UmRSA software (RSA Biomedical, Umeå, Sweden). In the RSA and OTS systems, Euler angles are used to express joint angles. All joint angles were calculated in the same order: flexion/extension, internal/external rotation, and varus/valgus, respectively. In this and our previous evaluations of knee rotations, we have used the femur as a fixed reference segment and the tibia as the moving segment (Uvehammer et al. 2000, Brandsson et al. 2002, Saari et al. 2004 a, b).

Hip-, knee-, and ankle-joint kinematics were calculated on the basis of positions derived from the 15 skin markers. We used a modified Coda pelvis (Bell et al. 1990) to define the pelvic segment and calculate hip-joint centers. The modification consisted of a reduction in the 2 posterior markers on the left and right posterior superior iliac spine (PSIS) into 1 marker on the sacrum, positioned at the mid-point between the left and right PSIS. 1 marker on the left and right anterior superior iliac spine completed the pelvic segment. Markers on the lateral knee-joint line, tibial tubercle, and lateral malleolus served as landmarks for the shank segment. The knee joint and ankle joint were defined as landmarks belonging to the shank segment. Furthermore, the distance between the lateral knee-joint line marker and the lateral malleolus marker was used as a scaling factor for intra-individual adjustments of knee- and ankle-joint placements. Finally, a foot segment was made, based on the 3 markers placed on the insertion of the Achilles tendon, lateral malleolus, and the dorsal surface of the metatarsal head. Before further calculations, we filtered all the marker position data using a Butterworth low-pass filter with a cut-off frequency of 15 Hz. Euler angles, expressed as joint angles, were calculated using Visual 3-D Professional software version 3.99.25.8 (C-Motion Inc., Germantown, MD), consistently using the proximal segment as the reference segment. We calculated all joint angles with Cardanic sequence (Woltring 1994), with a calculation order of X-Z-Y, i.e. flexion/extension, internal/external rotation, varus/valgus angulations, in order to comply with the order of calculation used in the RSA system.

### Statistics

Data from the RSA system were interpolated at 5-degree intervals using a linear approach. Corresponding values from the OTS system were then extracted for comparison. For the comparison and interpretation of data on a group basis, scatter plots with linear regression with a 95% CI were used. SPSS software version 17.0 was used for all statistical calculationsEthics

Informed consent was obtained from all subjects and the study was approved by the regional ethics committee in Göteborg, Sweden (R 301-99).

## Results

**Figure 1.**
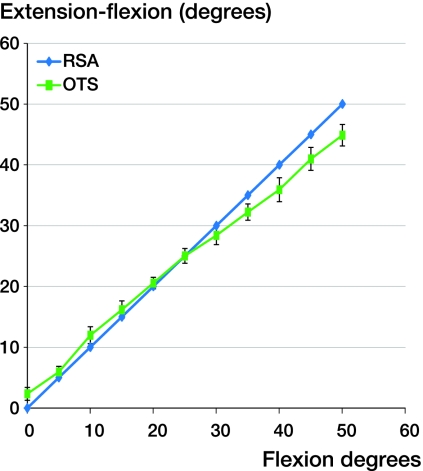
Mean values and SEM of flexion-extension angle for subjects as a group.

**Figure 2.**
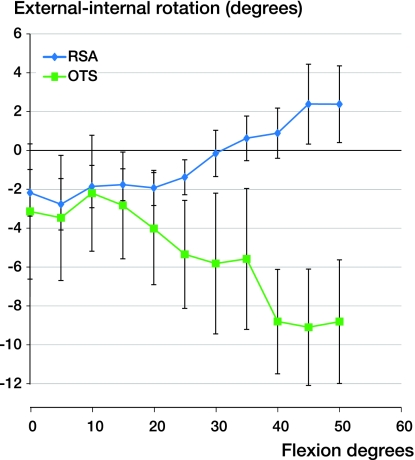
Mean values and SEM of internal-external rotation angle for subjects as a group.

**Figure 3.**
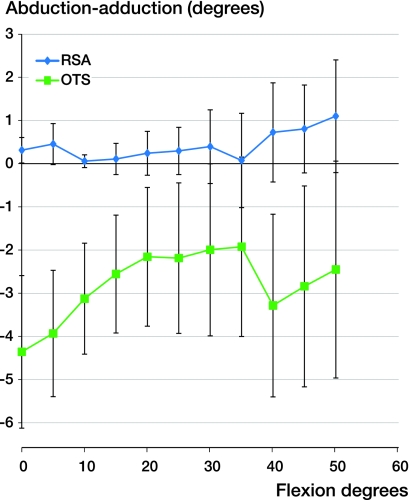
Mean values and SEM of abduction-adduction angle for subjects as a group.

**Figure 4.**
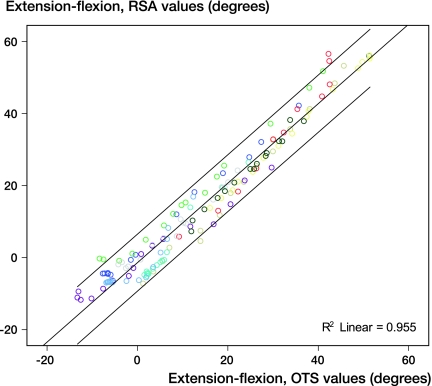
Scatter plot showing extension-flexion for 9 subjects (10 knees in total). Linear regression line and 95% CI. (R^2^ = 0.96).

**Figure 5.**
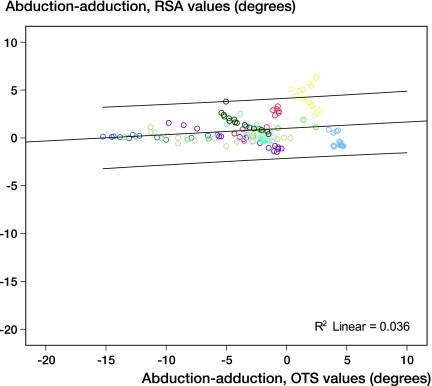
Scatter plot showing abduction-adduction for 9 subjects (10 knees in total). Linear regression line and 95% CI. (R^2^ = 0.04).

**Figure 6.**
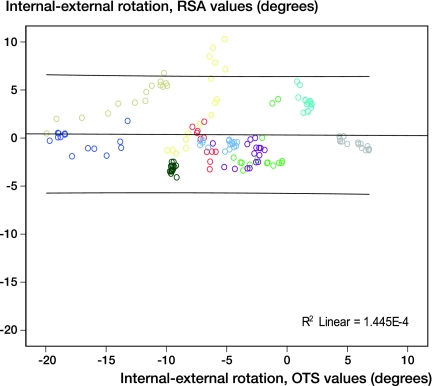
Scatter plot showing internal-external rotation for 9 subjects (10 knees in total). Linear regression line and 95% CI. (R^2^ = 0.0001).

Regarding flexion/extension, there was good agreement between the 2 systems, with a slight overestimation by the OTS system, starting with a difference of 2° at 0° of knee flexion, and gently switching to a moderate underestimation, resulting in a difference of 5° at a more pronounced flexion value (Figure 1). The external (–) / internal (+) rotation angle showed fairly good correspondence during the first 15° of flexion, with a mean difference within 1°. However, an increasing divergence was observed from 20° of flexion, ending in a maximum difference of 11° at 50° of knee flexion (Figure 2). Finally, for abduction (–)/adduction (+), a systematic difference with a variation of 2–4° was present throughout the measured range of motion (Figure 3). Linear regression analysis showed a strong correlation between extension-flexion angles as recorded with the 2 methods (R^2^ = 0.96) (Figure 4). However, data for abduction-adduction (–/+) and external-internal (–/+) rotation showed a poor correlation (R^2^ = 0.04 and R^2^ = 0.0001, respectively) (Figures 5 and 6).

## Discussion

We studied the 3-D kinematics of the knee with a motion capture system consisting of 8 cameras and a marker model based on 15 skin markers. We used simultaneous recording with a dynamic RSA, which served as the gold standard. To our knowledge, no such study has been performed previously. Examination of tibial internal/external rotation and abduction/adduction during extension of the knee has been studied with RSA in normal knees and in patients operated with different designs of total knee arthroplasty (Jonsson and Kärrholm 1994, 1999, Kärrholm et al. 1994, Uvehammer 2001, Brandsson et al. 2002, Saari et al. 2003, Weidow et al. 2007). In normal knees, these studies showed continuing external tibial rotation (mean 5–10 degrees from 40 to 0 degrees of extension) and a few degrees of adduction with proceeding extension. In the prosthetic designs studied, the amount of external rotation was smaller, was absent, or was reversed to internal tibial rotation during knee extension. Most designs showed, as in normal knees, tibial adduction with increasing extension, but usually with a tendency of more pronounced excursions.

As in the above-mentioned studies, our subjects started their motion from a flexed position and performed a weight-bearing knee extension. This movement is most similar to parts of stair climbing and cannot be fully compared to the motion pattern that occurs during walking. It is instead a standardized way to obtain recordings involving as much motion as possible, using dynamic RSA as applicable in our set-up.

Using our skin-marker model, flexion-extension could be measured with great accuracy using RSA data as a reference. Beyond 20 degrees of flexion, the first system tended to underestimate the magnitude of knee flexion. The true reason for this divergence is unclear. Small differences in the alignment of the coordinate axis to the body axes, resulting in so-called “crosstalk”, could be one reason. Increasing deformation of the soft tissues, resulting in increasing displacement of the skin with increasing flexion, could be another.

For small rotation around the longitudinal axis, the two systems recorded fairly uniform mean values. Past 20 degrees of flexion, we observed a systematic error. This error increased as the degree of flexion in the knee increased. It is probably not only related to the problem of soft-tissue motion but also an artifact of misalignment of the knee-flexion axis. This type of error typically results in a substantial increase in and a large degree of flexion error. Finally, the magnitude of external/internal rotation recorded was small, which means that the relative influence of an error will be greater.

Regarding abduction-adduction based on group values, a divergence was seen at the beginning and end of the measurement, with the best agreement between 20 and 35 degrees of flexion. The observed differences are probably caused by soft-tissue artifacts that become more pronounced when patients activate their muscles to initiate the extension. Furthermore, when they reach the end of an extension, they have to balance on one leg and compensate with hip and ankle movements to maintain knee stability. The artifacts caused by underlying soft tissues are probably so pronounced that they totally obscure the small movements that actually occur. Interestingly, we found a systematic difference between the methods. The OTS system consistently showed more abduction than the RSA system, which may be due to the fact that the knee-joint center in our marker model is not perfectly aligned with the RSA system. Furthermore, this offset may also be an effect of incorrect determination of the hip-joint and knee-joint center which, in the final calculation, are only defined by 1 virtual landmark each. Since the comparisons on an individual basis showed no correlation at all, it appears that the noise caused by the soft-tissue movement is far too high for any relevant analysis of abduction/adduction during a step-up.

Another limitation of our study is that only patients with TKA were included. A number of previous studies have shown that knees of this kind show a different pattern of internal/external rotation and abduction/adduction than normal knees. It might also be that due to a uniform and semi-constrained design, these knees move in a more consistent way, with less intra- and inter-individual variation than normal knees. Like normal knees, they did, however, display internal/external rotation and abduction/adduction movements with flexion of a magnitude that was not substantially different to that of normal knees, allowing a comparative methodological analysis.

2 major factors contribute to the data scatter presented in Figures 5 and 6. One factor is an inter-individual variability of the movement. The other factor is related to the resolution of the method used. In our study, the first type of error is the same for both methods and could be assumed to be a substantial part of the error observed for the RSA technique. Since the methodological error of RSA is small, the inherent amount of data scatter caused by the OTS system itself might be reflected to a certain extent by the difference between the error bars for the 2 methods when considering the size of the population under study. The question of whether this difference can be reduced further by using more skin markers, or different marker models, remains to be examined.

To increase the accuracy of the recordings based on external markers, we suggest that virtual markers could be added to our model. These markers should be set by a digitizer at the medial and lateral femur condyle. Further improvements to our model in order to evaluate the camera recordings of skin-marker positions could include firmly ensuring that the 2 local laboratory coordinate systems really do coincide, by simultaneously calibrating the 2 systems used to record joint motions. This was not possible in our study, however, since all the skin markers were not visible during the calibration procedure of the RSA system. Another way to address this issue would be to consider using a functional joint-center method (Halvorsen et al. 1999, Schwartz and Rozumalski 2005). These centers can also be computed with the RSA method. However, when it comes to the skin-marker method, at least 2 additional markers will be required on the thigh, which might raise concerns about new errors.

In summary, we found that the OTS system, together with this 15 skin-marker model, recorded flexion/extension with sufficient accuracy to allow one to study range of motion during walking. When it came to internal/external rotation—and above all, movements in the frontal plane—it appears that the noise caused by soft-tissue movements and perhaps also coordinate system misalignment are too high for meaningful analysis in this patient population. The same problem will probably appear during walking.
